# A Pan-Cancer Analysis of the Oncogenic Role of *BCL7B*: A Potential Biomarker for Prognosis and Immunotherapy

**DOI:** 10.3389/fgene.2022.906174

**Published:** 2022-07-15

**Authors:** Dinglong Yang, Hetong Li, Yujing Chen, Chunjiang Li, Weiping Ren, Yongbo Huang

**Affiliations:** ^1^ Second Clinical Medical College, Shanxi Medical University, Taiyuan, China; ^2^ School of Public Health, Xi’an Jiaotong University, Xian, China; ^3^ Department of Orthopedics, The Second Hospital of Shanxi Medical University, Taiyuan, China

**Keywords:** BCL7B, TCGA, pan-cancer, prognosis, immune infiltration, bioinformatics

## Abstract

**Background:** Previous studies have partly explored the role of B-cell CLL/lymphoma 7 protein family member B (*BCL7B*) in tumorigenesis and development. However, the prognosis and immunoregulatory value of *BCL7B* in pan-cancer patients remains unclear.

**Methods:** Through The Cancer Genome Atlas (TCGA) and Genotype-Tissue Expression (GTEx) databases, the distinct expression of *BCL7B* gene in 33 tumors and adjacent normal tissues was analyzed. The Kaplan–Meier method (univariate Cox regression analysis and Kaplan–Meier curve) was used to identify the cancer types whose *BCL7B* gene expression was related to prognosis. The receiver operating characteristic (ROC) curve was used to elucidate the diagnosis value of *BCL7B* gene. Spearman’s rank correlation coefficient was used to explore the relationship between *BCL7B* gene expression and immune cell infiltration, immune checkpoints, DNA methylation, DNA repair genes, immune-activating genes, immune-suppressing genes, immune subtypes, tumor mutation burden (TMB), and microsatellite instability (MSI). The Wilcoxon rank sum test and Kruskal–Wallis test were used to compare the expression of *BCL7B* gene in tumor tissues with different clinicopathological features. Gene set enrichment analysis (GSEA) was conducted to identify the tumor-related pathways in pan-cancer. The Human Protein Atlas (HPA) database was used to verify the *BCL7B* gene expression at the protein level.

**Results:** High expression of *BCL7B* was associated with an inferior prognosis in glioblastoma multiforme (GBM), glioma (GBMLGG), kidney chromophobe (KICH), brain lower grade glioma (LGG), oral squamous cell carcinoma (OSCC), rectum adenocarcinoma (READ), and uveal melanoma (UVM). Low expression of *BCL7B* was associated with a poor prognosis in kidney renal clear cell carcinoma (KIRC), kidney renal papillary cell carcinoma (KIRP), skin cutaneous melanoma (SKCM), thyroid carcinoma (THCA), and sarcoma (SARC). The *BCL7B* gene expression had varying degrees of correlation with 24 immune cell subsets in 37 tumor environments such as adrenocortical carcinoma (ACC) and bladder urothelial carcinoma (BCLA). Spearman’s rank correlation coefficient showed that *BCL7B* gene expression had different degrees of correlation with 47 immune checkpoints, 46 immune-activating genes, 24 immune-suppressing genes, 5 DNA repair genes, and DNA methylation, TMB, and MSI in 39 tumors. GSEA suggested that *BCL7B* was notably associated with cancer-related and immune-related pathways.

**Conclusion:** In summary, *BCL7B* gene has a high diagnostic and prognostic value in pan-cancer and is related to the infiltration of 24 immune cell subsets in pan-cancer.

## Introduction

Cancer is the second disease leading to death behind cardiovascular diseases (Myerson et al., 2019). Cancer remains a devastating disease, and the increasing cancer-related deaths have a huge impact on public health, which has attracted wide notice (Lamoral-Theys et al., 2010). A great number of researches have been conducted to understand the development and related mechanisms of cancers (Torre et al., 2015). Although we have made big progress in the treatment of certain cancers, most cancer patients have a poor prognosis. Cancer metastatic, dissemination, and relapse are critical determinants of prognosis, but the underlying mechanisms for malignancy remain unknown (Chaffer et al., 2013; Peng et al., 2015). Therefore, a biomarker is urgently needed to forecast cancer prognosis and improve cancer therapy. Immune system plays a crucial role in controlling cancer, making full use of the immune system to eliminate cancer, and has a great potential (Lee et al., 2016). However, immune evasion is a major barrier to successful cancer immunotherapy (Zeng et al., 2020; Zhang et al., 2020). Many immune evasion mechanisms have been identified, including immune checkpoint blockade (ICB), that help immune system to recognize and attack cancer cells (Jiang et al., 2018). The human cancer immune microenvironment is the key to understand the immunity in response to tumor immunotherapy and tumor progression (Wang et al., 2016).

*BCL7B*, also called B-cell CLL/lymphoma 7 protein family member B, is a member of the BCL7 family including BCL7A, BCL7B, and BCL7C proteins. The BCL7 family was first discovered when B-cell CLL/lymphoma 7 protein family member A (BCL7A) was found to be involved in the complex translocation of a Burkitt lymphoma cell line (Zani et al., 1996). It is found that BCL7 family members play a crucial role in the progression of several cancers. Previous studies have proved that increased BCL7A protects from Burkitt lymphoma, astrocytoma, cutaneous T-cell lymphoma, B-cell non-Hodgkin’s lymphoma, and osteosarcoma (Zani et al., 1996; van Doorn et al., 2005; Potter et al., 2008; Morton et al., 2009; Dai et al., 2021). *BCL7B*, a member of BCL7 family, also plays a critical role in tumorigenesis and development (Mathies et al., 2017). *BCL7B* was found as a predictor of poor prognosis of pancreatic cancers, and it promotes cell motility and invasion by influencing CREB signaling (Taniuchi et al., 2018). In postoperative pancreatic cancer patients, overexpression of *BCL7B* accurately predicted the poor prognosis compared to the TNM staging system (Taniuchi et al., 2019). *BCL7B* knockdown induced nuclear enlargement which suppresses cell death and promotes the multinuclei phenotype in KATOIII human gastric cancer cells (Uehara et al., 2015). *BCL7B* also contributes to non-neoplastic diseases such as alcohol dependence (Mathies et al., 2017). It was reported that *BCL7B* is deleted in the patients with Williams–Beuren syndrome, and the malignant diseases occurring in Williams–Beuren syndrome patients are related to *BCL7B* aberration (Uehara et al., 2015). In addition, *BCL7B* is involved in immune regulation. Our previous study found that *BCL7B* was associated with immune infiltration in sarcoma, which was consistent with this study (Yang et al., 2021). Considering the limited reports of *BCL7B* gene in different kinds of cancers, a comprehensive analysis of *BCL7B* gene is necessary to explore effective prognostic biomarkers and immune-related mechanisms in cancers.

In this study, we used specific data to compare the *BCL7B* gene expression in different kinds of tumors with the corresponding adjacent normal tissues. Our results showed that *BCL7B* was a potential diagnostic and prognostic biomarker in multiple cancer such as glioblastoma multiforme (GBM) and oral squamous cell carcinoma (OSCC). Also, we explored the potential signaling pathways that *BCL7B* gene participates in tumorigenesis, development, and tumor microenvironment. Compared with the existing *BCL7B* gene-related experiments, our work systematically and comprehensively studied the functional role of *BCL7B* gene in pan-cancer, highlighting its prognostic and diagnostic value, and potential mechanism in cancers.

## Materials and Methods

### Data Acquisition

The RNA-seq data of 33 tumor and adjacent normal tissues were extracted from The Cancer Genome Atlas (TCGA) database (https://tcgadata.nci.nih.gov/tcga/). Since TCGA database mainly collected tumor tissues, we also downloaded normal and tumor RNA-seq data from the Genotype-Tissue Expression (GTEx) dataset. To ensure more reliable results, the data from TCGA and GTEx databases was combined for expression analysis. Specifically, the RNA-seq data of osteosarcoma were extracted from the Therapeutically Applicable Research To Generate Effective Treatments (TARGET) (https://ocg.cancer.gov/programs/target) database. Then, RNA-seq was transformed into TPM (transcripts per million reads) for the following analysis. The TPM data in log2 format were used to analyze the *BCL7B* gene expression in normal and tumor tissues.

### The Protein Expression of *BCL7B* in the Human Protein Atlas Database

The Human Protein Atlas (HPA: https://www.proteinatlas.org/) database was used to explore the protein expression of *BCL7B* in different tissues. Then, we downloaded the immunohistochemical staining images from the HPA database to verify *BCL7B* expression of tumor and corresponding normal tissues in TCGA and GTEx databases.

### Diagnosis and Prognosis Analysis

The prognostic difference of the *BCL7B* gene expression group in 40 kinds of tumors was analyzed with the dichotomy method (Liu et al., 2018). The survival R package (version 3.2–10) was used to analyze the survival differences between the low- and high-expression group of *BCL7B* in tumors, and the survminer R package (version 0.4.9) was used for visualization. The Kaplan–Meier method (univariate Cox regression analysis and Kaplan–Meier curve) was used to elucidate the follow-up duration including over survival (OS), disease-specific survival (DSS), and progress-free interval (PFI). Furthermore, to explore the diagnostic value of *BCL7B* in multiple cancers, the pROC R package (version 1.17.0.1) was used for statistical analysis, and ggplot2 R package (version 3.3.3) was used to make the receiver operating characteristic (ROC) curve. The ROC curves of *BCL7B* with area under the Curve (AUC) more than 0.8 exhibited high diagnostic values in different kinds of cancers.

### Relationship Between *BCL7B* Expression and Immunity

We used RNA-Seq expression profile data to explore the infiltration of 24 immune cells into tumor tissues. The GSVA R package (version 1.34.0) was used to analyze the relationship between *BCL7B* expression and immune cell enrichment in 39 kinds of tumors (Hänzelmann et al., 2013). The estimate R package (version 1.0.13) was used to explore the correlation between *BCL7B* expression and stromal score, immune score, and estimate score. The 24 kinds of immune cells included dendritic cells (DC), activated DC (aDC), B cells, CD8+T cells, cytotoxic cells, eosinophils, immature DC (iDC), macrophages, mast cells, neutrophils, natural killer (NK) cells, NK CD56^+^ cells, NK CD56^−^cells, plasmacytoid DC (pDC), T cells, T helper cells, T central memory (Tcm), T effector memory (Tem), T follicular helper (Tfh), T gamma delta (Tgd), Th1 cells, Th17 cells, Th2 cells, and regulatory T (Treg) cells (Bindea et al., 2013). Furthermore, we extracted 47 immune checkpoints, 46 immune-activating genes, 24 immune-suppressing genes, and 5 DNA repair genes. Spearman’s rank correlation coefficient was used to analyze the relationship between *BCL7B* expression and immune checkpoints, immune-activating genes, immunosuppressive status-related genes, and DNA repair genes in 40 tumors. The ggplot2 R package (version 3.3.3) was used for visualization. The threshold values were considered as follows: ∗*p* < 0.05 indicates a general correlation, and ∗∗*p* < 0.01 indicates a high correlation.

### Relationship Between *BCL7B* Expression and DNA Methylation, TMB, and MSI

Spearman’s rank correlation coefficient was used to analyze the relationship between *BCL7B* expression and DNA methylation, tumor mutation burden (TMB), and microsatellite instability (MSI). DNA methylation analysis was based on Illumina methylation 450 data and cg27441048 probe. The ggplot2 R package (version 3.3.3) was used for visualization. The threshold values were considered as follows: ∗*p* < 0.05 indicates a mild correlation, ∗∗*p* < 0.01 indicates a moderate correlation, and ∗∗∗*p* < 0.001 indicates a high correlation.

### Gene Set Enrichment Analysis

According to the gene expression matrix, GSEA (http://software.broadinstitute.org/gsea/msigdb/index.jsp) can predict gene-related signaling pathways and phenotypes by analyzing enrichment differences of functions and pathways between the high- and low-expression group of *BCL7B* in 33 kinds of tumors (Subramanian et al., 2005). In this study, the R package clusterProfiler (3.14.3) was used to conduct GSEA of *BCL7B* gene in tumors with low and high expression (Yu et al., 2012). The adjusted *p*-value (<0.05), normalized enrichment score (|NES| > 1), and FDR q value (<0.25) were used to classify enrichment differences of function in each phenotype.

### Immune Subtype Association

Associations between *BCL7B* expression and immune subtypes across human pan-cancer were analyzed using the TISIDB database.

### Statistical Analysis

All statistical analyses were carried out using R (version 3.6.2). The Shapiro–Wilk test was used for the normality test. The Wilcoxon rank sum test and Kruskal–Wallis test were used to compare the expression of *BCL7B* in tissues with different clinicopathological features. Univariate Cox regression analysis was used to elucidate the follow-up duration including OS, DSS, and PFI. In all tests, the hypothetical test was two-sided, and *p*-value <0.05 was regarded as statistically significant. The threshold values were considered as follows: ∗*p* < 0.05, ∗∗*p* < 0.01, ∗∗∗*p* < 0.001, and ns, *p* ≥ 0.05.

## Results

### The Expression Levels of *BCL7B*

**FIGURE 1:**
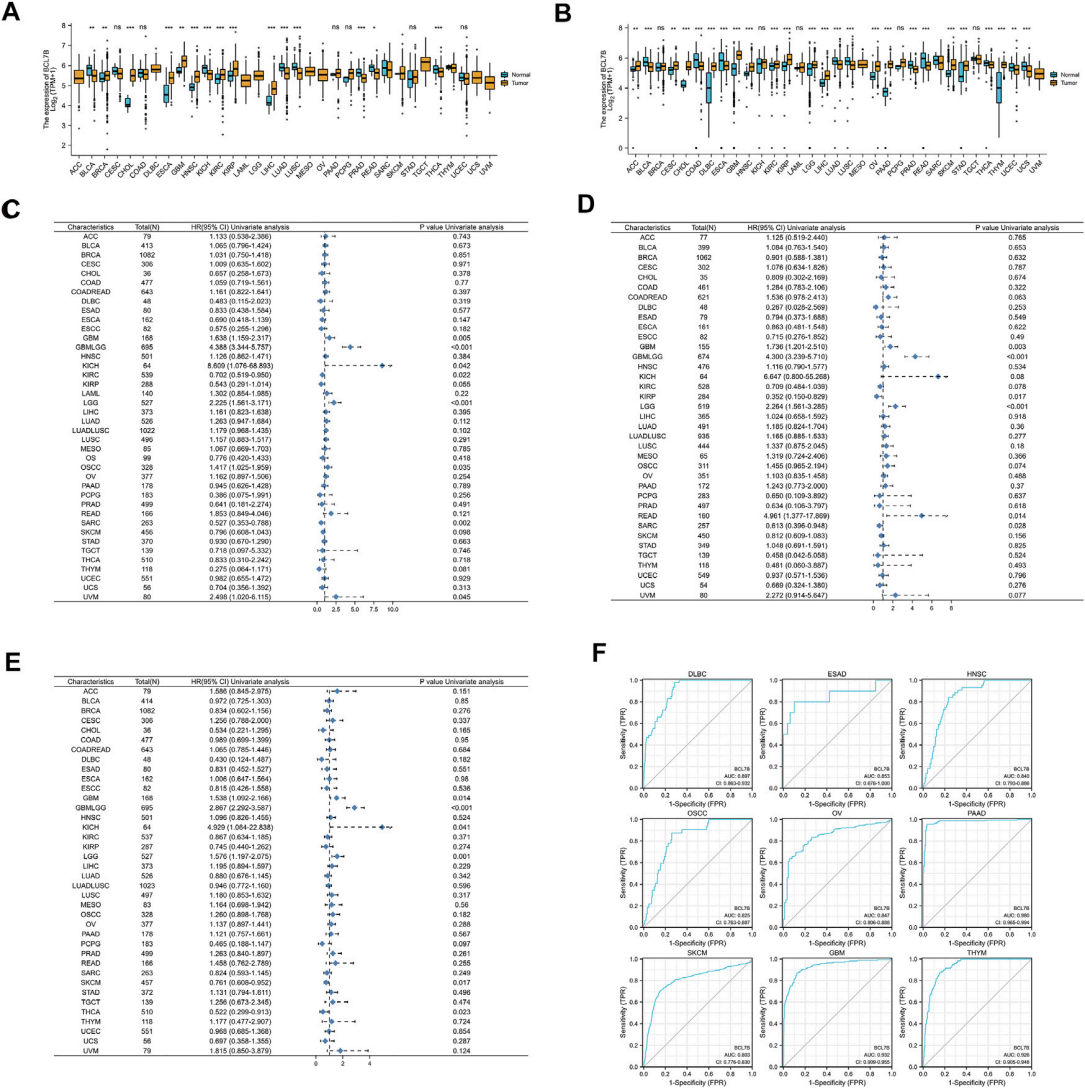
Expression of *BCL7B* gene, and prognostic and diagnostic value in pan-cancer. **(A)** Expression of *BCL7B* gene in 33 types of cancers based on the data from TCGA database. **(B)** Expression of *BCL7B* gene in 33 types of cancers based on the data from TCGA and GTEx databases. **(C)** OS showed that *BCL7B* was notably correlated with the prognosis of GBM (*p* = 0.014), GBMLGG (*p* < 0.001), KICH (*p* = 0.041), KIRC (*p* = 0.022), LGG (*p* = 0.001), OSCC (*p* = 0.035), SARC (*p* = 0.002), and UVM (*p* = 0.045). **(D)** DSS displayed that *BCL7B* is markedly related with the prognosis of GBM (*p* = 0.003), GBMLGG (*p* < 0.001), KIRP (*p* = 0.017), LGG (*p* < 0.001), READ (*p* = 0.014), and SARC (*p* = 0.028). **(E)** PFI reflected that *BCL7B* was dramatically correlated with the prognosis of GBM (*p* = 0.014), GBMLGG (*p* < 0.001), KICH (*p* = 0.041), LGG (*p* = 0.001), SKCM (*p* = 0.017), and THCA (*p* = 0.023). **(F)** ROC curves showed that *BCL7B* had a high diagnostic value (AUC>0.8) in DLBC, ESAD, HNSC, OSCC, OV, PAAD, SKCM, GBM, and THYM. ∗*p* < 0.05, ∗∗*p* < 0.01, ∗∗∗*p* < 0.001, and ns, *p* ≥ 0.05.

Compared with adjacent normal tissues, the analysis of TCGA dataset showed that the *BCL7B* gene showed a low expression in bladder urothelial carcinoma (BLCA), kidney chromophobe (KICH), lung adenocarcinoma (LUAD), lung squamous cell carcinoma (LUSC), prostate adenocarcinoma (PRAD), rectum adenocarcinoma (READ), and thyroid carcinoma (THCA). Breast invasive carcinoma (BRCA), cholangiocarcinoma (CHOL), esophageal carcinoma (ESCA), GBM, head and neck squamous cell carcinoma (HNSC), kidney renal clear cell carcinoma (KIRC), kidney renal papillary cell carcinoma (KIRP), and liver hepatocellular carcinoma (LIHC) had high *BCL7B* gene expression (Figure 1A). Furthermore, to get more convincing results, we also combined data from TCGA and GTEx databases for the expression analysis. As showed in Figure 1B, *BCL7B* gene expression was low in BCLA, colon adenocarcinoma (COAD), ESCA, LUAD, LUSC, PRAD, READ, THCA, uterine corpus endometrial carcinoma (UCEC), and uterine carcinosarcoma (UCS). The *BCL7B* gene expression was high in adrenocortical carcinoma (ACC), cervical squamous cell carcinoma and endocervical adenocarcinoma (CESC), CHOL, lymphoid neoplasm diffused large B-cell lymphoma (DLBC), GBM, HNSC, KIRC, KIRP, brain lower grade glioma (LGG), LIHC, ovarian serous cystadenocarcinoma (OV), pancreatic adenocarcinoma (PAAD), skin cutaneous melanoma (SKCM), stomach adenocarcinoma (STAD), and thymoma (THYM).

We also explored the protein level of *BCL7B*. Using the HPA database, we found that the protein level of *BCL7B* was the highest in testis (Supplementary Figure 1A). In tumor tissues, the *BCL7B* protein level was the highest in testis cancer and lowest in endometrial cancer (Supplementary Figure 1B). Furthermore, the protein location of *BCL7B* was mainly in the nucleoplasm (Supplementary Figure 1C).

### The Prognostic Value of *BCL7B* in Tumors

**FIGURE 2:**
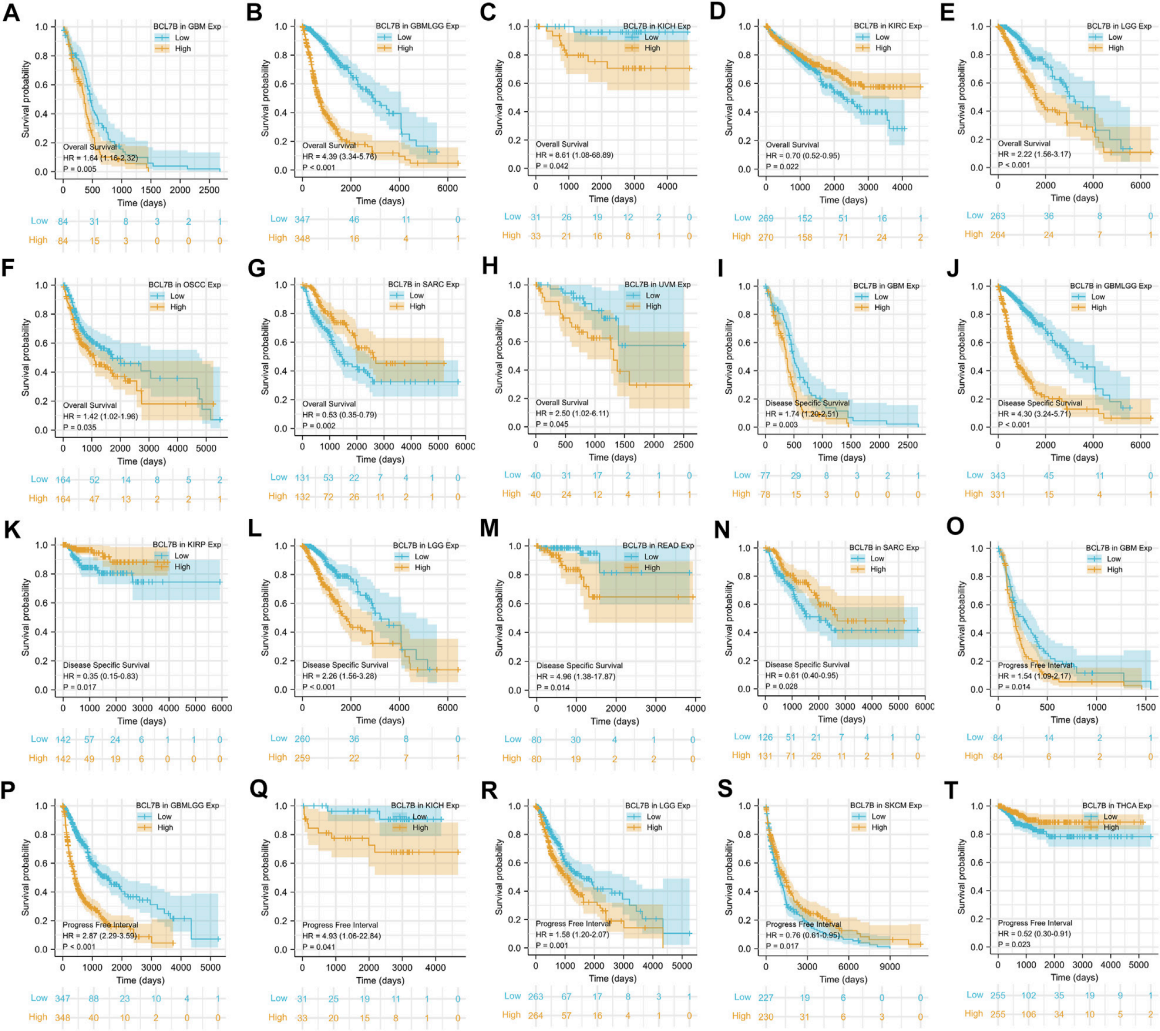
Survival curves of cancer types with significant correlation between *BCL7B* gene expression and prognosis. High expression of *BCL7B* was associated with an inferior prognosis in GBM (*p* = 0.005) **(A,I,O)**, GBMLGG (*p* < 0.001) **(B,J,P)**, KICH (*p* = 0.042) **(C,Q)**, LGG (*p* < 0.001) **(E,L,R)**, OSCC (*p* = 0.035) **(F)**, READ (*p* = 0.014) **(M)**, and UVM (*p* = 0.045) **(H)**. Low expression of *BCL7B* was associated with a poor prognosis in KIRC (*p* = 0.022) **(D)**, KIRP (*p* = 0.017) **(K)**, SKCM (*p* = 0.017) **(S)**, THCA (*p* = 0.023) **(T)**, and SARC (*p* = 0.002) **(G,N)**.

To determine the correlation of *BCL7B* gene expression with the patient prognosis in 40 tumors, we used gene expression profile data and univariate Cox regression analysis to draw forest plots. OS showed that *BCL7B* was significantly related to the prognosis of GBM (*p* = 0.014), glioma (GBMLGG, *p* < 0.001), KICH (*p* = 0.041), KIRC (*p* = 0.022), LGG (*p* = 0.001), OSCC (*p* = 0.035), sarcoma (SARC, *p* = 0.002), and uveal melanoma (UVM, *p* = 0.045) (Figure 1C, Figures 2A–H). DSS displayed that *BCL7B* was notably correlated with the prognosis of GBM (*p* = 0.003), GBMLGG (*p* < 0.001), KIRP (*p* = 0.017), LGG (*p* < 0.001), READ (*p* = 0.014), and SARC (*p* = 0.028) (Figure 1D). PFI reflected that *BCL7B* was markedly correlated with the prognosis of GBM (*p* = 0.014), GBMLGG (*p* < 0.001), KICH (*p* = 0.041), LGG (*p* = 0.001), SKCM (*p* = 0.017), and THCA (*p* = 0.023) (Figure 1E). These outcomes showed that low expression of *BCL7B* was related to the low survival rate of KIRC, SARC, KIRP, SKCM, and THCA (Figures 2D,G,K,N,S,T), and high expression of *BCL7B* was related to the low survival rate of GBM, GBMLGG, KICH, LGG, OSCC, UVM, and READ (Figures 2A–C, E,F, H–J, L,M, O–R). The abovementioned results reflected that *BCL7B* was a risk and protective factor in multiple cancer patients.

### The Diagnostic Value of *BCL7B* in Tumors

The ROC curve was depicted to explore the diagnostic value of *BCL7B* in 34 tumors. ROC curves showed that *BCL7B* gene had a high diagnostic value (AUC>0.8) in DLBC (AUC = 0.897), esophageal adenocarcinoma (ESAD, AUC = 0.853), HNSC (AUC = 0.840), OSCC (AUC = 0.825), OV (AUC = 0.847), PAAD (AUC = 0.980), SKCM (AUC = 0.803), GBM (AUC = 0.932), and THYM (AUC = 0.926) (Figure 1F).

### Relationship Between *BCL7B* and 24 Types of Infiltrating Immune Cells

**FIGURE 3:**
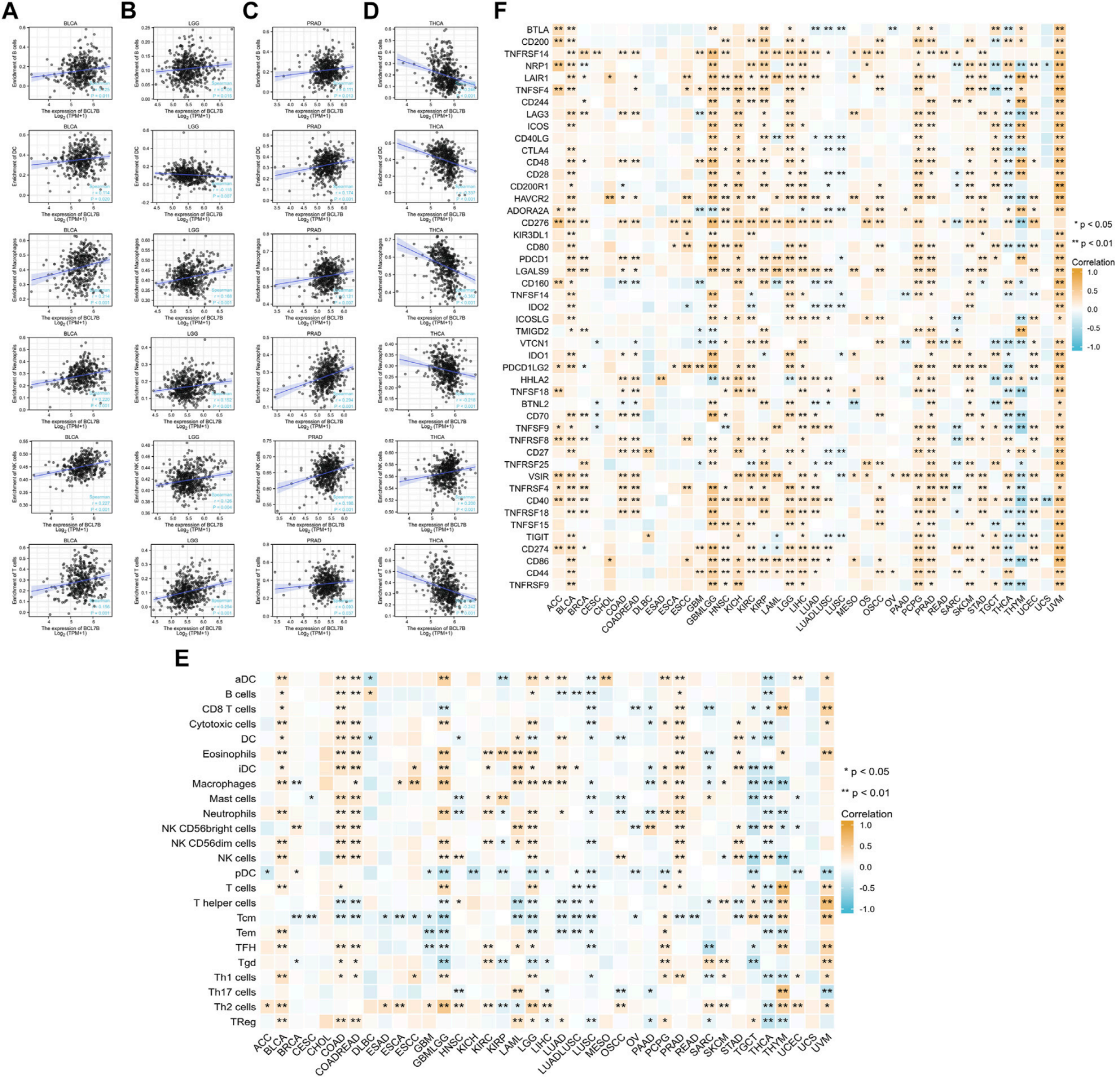
Correlation analysis of *BCL7B* gene expression in immune cell infiltration and 47 immune checkpoints. **(A–D)** In 39 types of tumors, only BLCA, LGG, PRAD, and THCA had correlation with all six immune cells including B cells, dendritic cells, neutrophils, T cells, macrophages, and NK cells **(E)** Correlation between *BCL7B* gene expression and infiltration of 24 immune cells in 39 types of tumors. **(F)** Relationship between *BCL7B* gene expression and 47 immune checkpoints in 39 types of tumors. ∗*p* < 0.05 and ∗∗*p* < 0.01.

The correlation between the immune infiltration level and expression level (TPM) of *BCL7B* was analyzed by Spearman’s correlation in 39 kinds of tumor environment. We first analyzed the relationships between *BCL7B* gene expression and six types of infiltrating immune cells (B cells, dendritic cells, neutrophils, T cells, macrophages, and NK cells). We found that only BLCA, LGG, PRAD, and THCA had correlation with all the abovementioned six immune cells. The outcomes indicated that *BCL7B* gene expression was positively correlated with the expression of B cells (*p* = 0.011), dendritic cells (*p* = 0.020), neutrophils (*p* < 0.001), T cells (*p* = 0.001), macrophages (*p* < 0.001), and NK cells (*p* < 0.001) in BLCA (Figure 3A). The expression of *BCL7B* was negatively correlated with the expression of dendritic cells (*p* = 0.007) and positively with B cells (*p* = 0.015), neutrophils (*p* < 0.001), T cells (*p* < 0.001), macrophages (*p* < 0.001), and NK cells (*p* = 0.004) in LGG (Figure 3B). The results in PRAD and THCA are shown in Figures 3C,D. To make a more convincing result, we conducted a more in-depth research based on tumor samples. The results revealed that the *BCL7B* gene expression had different degrees of correlation with the infiltrating immune cell subsets in a multiple tumor environment, including ACC, BLCA, BRCA, CESC, COAD, COADREAD, DLBC, ESAD, ESCA, esophageal squamous cell carcinoma (ESCC), GBM, GBMLGG, HNSC, KICH, KIRC, KIRP, LAML, LGG, LIHC, LUAD, LUADLUSC, LUSC, mesothelioma (MESO), OSCC, OV, PAAD, pheochromocytoma and paraganglioma (PCPG), PRAD, READ, SARC, SKCM, STAD, testicular germ cell tumor (TGCT), THCA, THYM, UCEC, and UVM (Figure 3E).

**FIGURE 4:**
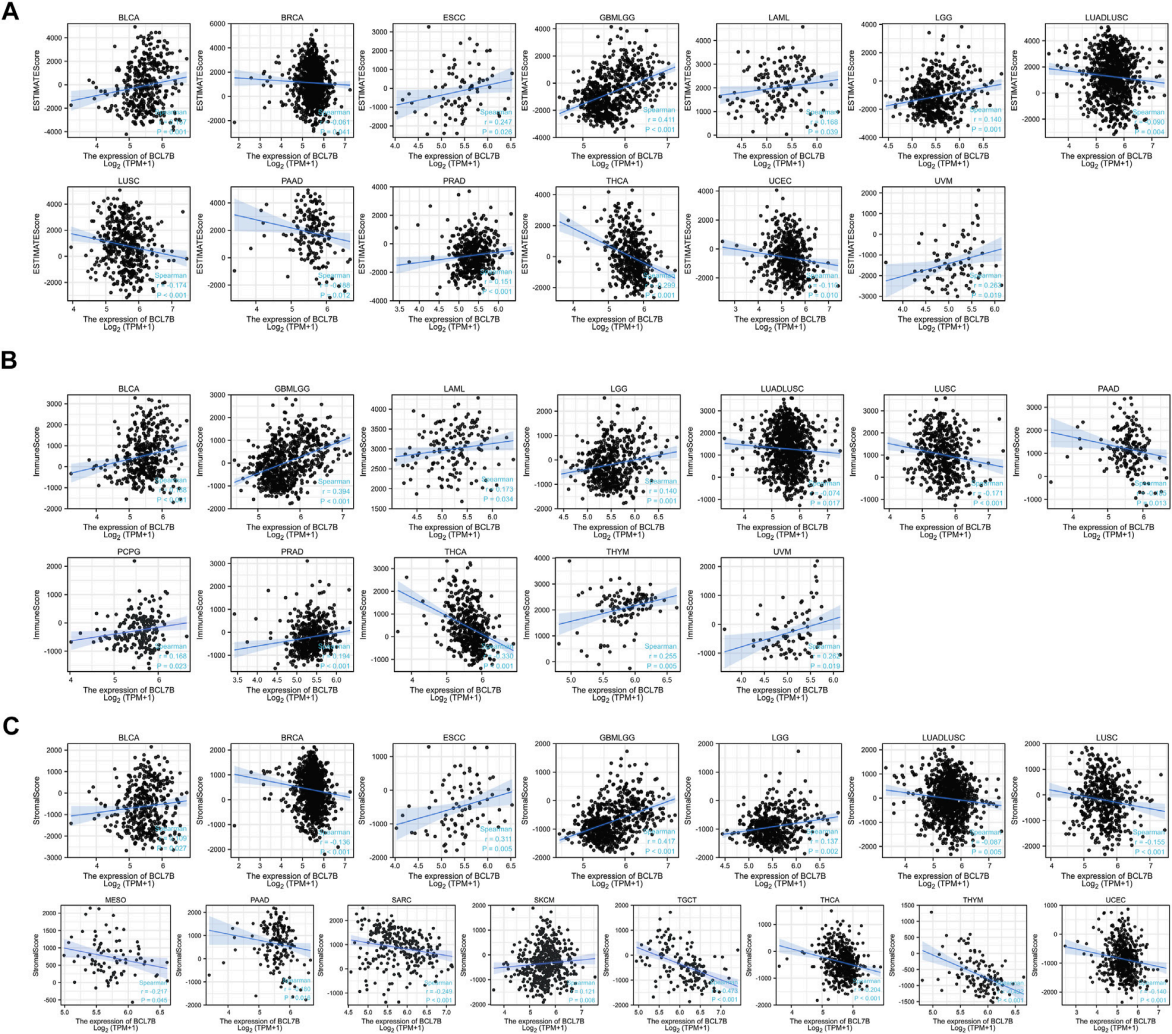
Relationship between *BCL7B* gene expression and estimate score, immune score, and stromal score in 39 kinds of tumors. **(A)** Expression of *BCL7B* gene in the ESTIMATE immune score concerned with BLCA, BRCA, ESCC, GBMLGG, LAML, LGG, LUADLUSC, LUSC, PAAD, PRAD, THCA, UCEC, and UVM was prominently correlated. **(B)** Expression of *BCL7B* gene in the immune score was concerned with BLCA, GBMLGG, LAML, LGG, LUADLUSC, LUSC, PAAD, PCPG, PRAD, THCA, THYM, and UVM that are markedly correlated. **(C)** Expression of *BCL7B* gene in the stromal score was concerned with BLCA, BRCA, ESCC, GBMLGG, LGG, LUADLUSC, LUSC, MESO, PAAD, SARC, SKCM, TGCT, THCA, THYM, and UCEC that are notably correlated.

To further explore the role of *BCL7B* in tumor microenvironment, our result reflected that *BCL7B* had different degrees of correlation with 47 immune checkpoints (Figure 3F). Furthermore, as shown in Figure 4A, we found that the expression of the *BCL7B* gene was correlated with ESTIMATEScore in BLCA (*p* = 0.001), BRCA (*p* = 0.041), ESCC (*p* = 0.026), GBMLGG (*p* < 0.001), LAML (*p* = 0.039), LGG (*p* = 0.001), LUADLUSC (*p* = 0.004), LUSC (*p* < 0.001), PAAD (*p* = 0.012), PRAD (*p* < 0.001), THCA (*p* < 0.001), UCEC (*p* = 0.010), and UVM (*p* = 0.019). The relationships between *BCL7B* gene expression and ImmuneScore (Figure 4B) and StromalScore (Figure 4C) were similar in multiple tumor samples.

### Relationship Between *BCL7B* Gene Expression and DNA Methylation, TMB, and MSI

**FIGURE 5:**
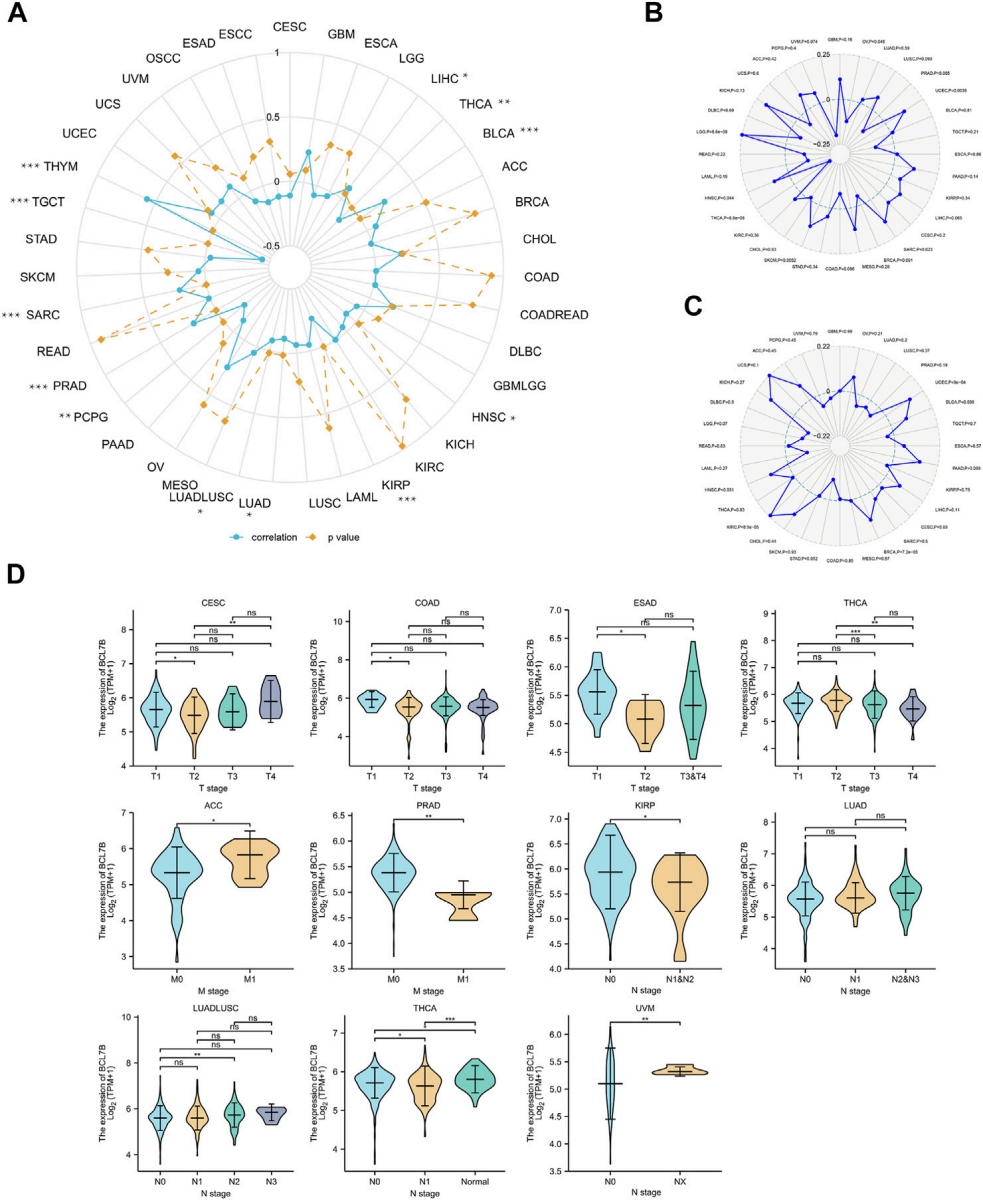
Relationship between *BCL7B* gene expression and DNA methylation, and clinical TNM stage in pan-cancer. **(A)** *BCL7B* gene expression was notably positively correlated with DNA methylation in LIHC (*p* = 0.049), BLCA (*p* < 0.001), PRAD (*p* < 0.001), SARC (*p* < 0.001), and THYM (*p* < 0.001) and negatively in THCA (*p* = 0.002), HNSC (*p* = 0.027), KIRP (*p* < 0.001), LUADLUSC (*p* = 0.016), LUAD (*p* = 0.017), PCPG (*p* = 0.005), and TGCT (*p* = 0.002). **(B)** sExpression of *BCL7B* gene was noticeably relevant to TMB in OV (*p* = 0.046), PRAD (*p* = 0.005), UCEC (*p* = 0.0036), SARC (*p* = 0.023), SKCM (*p* = 0.0052), THCA (*p* < 0.001), HNSC (*p* = 0.044), and LGG (*p* < 0.001). **(C)** Expression of *BCL7B* in UCEC (*p* < 0.001), BRCA (*p* < 0.001), KIRC (*p* < 0.001), and HNSC (*p* = 0.031) was notably correlated with MSI. **(D)** *BCL7B* gene expression was different in cancer patients with distinguishing clinical T (CESC, COAD, ESAD, and THCA), M (ACC and PRAD), and N (KIRP, LUADLUSC, THCA, and UVM) stages. ∗*p* < 0.05, ∗∗*p* < 0.01, and ∗∗∗*p* < 0.001.

Methylation, one form of DNA modification, affects the DNA transcription process without altering the DNA sequence (Fang et al., 2016). Based on Illumina methylation 450 data and cg27441048 probe, we explored the relationship between *BCL7B* gene expression and DNA methylation. The result revealed that the *BCL7B* gene expression was positively correlated with DNA methylation in BLCA (*p* < 0.001), LIHC (*p* = 0.049), PRAD (*p* < 0.001), SARC (*p* < 0.001), and THYM (*p* < 0.001) and negatively in HNSC (*p* = 0.027), KIRP (*p* < 0.001), LUAD (*p* = 0.017), LUADLUSC (*p* = 0.016), PCPG (*p* = 0.005), TGCT (*p* < 0.001), and THCA (*p* = 0.002) (Figure 5A; Table 1). In addition, the expression of *BCL7B* gene was noticeably relevant to TMB in OV (*p* = 0.046), PRAD (*p* = 0.005), UCEC (*p* = 0.0036), SARC (*p* = 0.023), SKCM (*p* = 0.0052), THCA (*p* < 0.001), HNSC (*p* = 0.044), and LGG (*p* < 0.001) (Figure 5B). The expression of *BCL7B* gene was notably related to MSI in UCEC (*p* < 0.001), BRCA (*p* < 0.001), KIRC (*p* < 0.001), and HNSC (*p* = 0.031) (Figure 5C).

**TABLE 1:** Relationship between *BCL7B* gene expression and DNA methylation (Illumina methylation 450 data and cg27441048 probe) in pan-cancer.

Characteristic	Correlation	*p*-value
ACC	0.075	0.510
BLCA	0.230	2.86E-06
BRCA	−0.007	0.838
CESC	−0.109	0.057
CHOL	0.209	0.221
COAD	0.007	0.909
COADREAD	0.013	0.790
DLBC	0.195	0.184
ESAD	−0.133	0.238
ESCA	−0.076	0.335
ESCC	−0.111	0.322
GBM	0.238	0.096
GBMLGG	−0.064	0.130
HNSC	−0.099	0.027
KICH	−0.048	0.702
KIRC	−0.002	0.973
KIRP	−0.236	8.70E-05
LAML	−0.048	0.617
LGG	−0.043	0.333
LIHC	0.102	0.049
LUAD	−0.112	0.017
LUADLUSC	−0.084	0.016
LUSC	−0.063	0.223
MESO	0.053	0.630
OSCC	−0.087	0.117
OV	0.250	0.595
PAAD	−0.117	0.120
PCPG	−0.208	0.005
PRAD	0.201	6.62E-06
READ	0.011	0.911
SARC	0.213	5.81E-04
SKCM	0.049	0.289
STAD	−0.041	0.450
TGCT	−0.441	2.37E-08
THCA	−0.135	0.002
THYM	0.570	1.32E-10
UCEC	0.077	0.112
UCS	0.075	0.580
UVM	0.117	0.301

### Relationship Between *BCL7B* Gene Expression and Clinicopathological Characteristics

We explored the relationship between *BCL7B* expression and clinicopathological characteristics including age, gender, and TMN stage. By dividing cancer patients into two groups based on the median *BCL7B* gene expression, we found that *BCL7B* gene expression was correlated with age in CHOL (*p* = 0.001), GBMLGG (*p* < 0.001), LAML (*p* = 0.027), LGG (*p* < 0.001), and THYM (*p* < 0.001) and correlated with gender in COADREAD (*p* = 0.043), HNSC (*p* = 0.007), LGG (*p* < 0.001), LIHC (*p* = 0.01), OSCC (*p* = 0.014), and SARC (*p* = 0.011) (Table 2). Furthermore, our result revealed that the expression of *BCL7B* was related to TMN stage in ACC, CESC, COAD, ESAD, KIRP, LUAD, LUADLUSC, PRAD, THCA, and UVM (Figure 5D).

**TABLE 2:** BCL7B expression∗ associated with age and gender of cancer patients in pan-cancer. * Categorical-dependent variables, greater or less than the median expression level.

Cancer	Age			Gender		
	Young (n)	Old (n)	*p*-value	Male (n)	Female (n)	*p*-value
ACC	41 (< = 50)	38 (>50)	0.602	31	48	0.32
BLCA	234 (< = 70)	180 (>70)	0.058	305	109	0.624
BRCA	601 (< = 60)	482 (>60)	0.298	-	-	-
CESC	188 (< = 50)	118 (>50)	0.112	-	-	-
CHOL	17 (< = 65)	19 (>65)	0.001	16	20	0.14
COAD	194 (< = 65)	284 (>65)	0.207	256	226	0.09
COADREAD	276 (< = 65)	368 (>65)	0.385	301	343	0.043
DLBC	27 (< = 60)	21 (>60)	0.245	22	26	0.859
ESAD	30 (< = 60)	50 (>60)	0.639	69	11	0.627
ESCA	83 (< = 60)	79 (>60)	0.337	139	23	0.754
ESCC	53 (< = 60)	29 (>60)	0.091	70	12	0.976
GBM	87 (< = 60)	81 (>60)	0.329	109	59	0.39
GBMLGG	553 (< = 60)	143 (>60)	<0.001	398	298	0.4
HNSC	245 (< = 60)	256 (>60)	0.693	368	134	0.007
KICH	33 (< = 50)	32 (>50)	0.944	39	26	0.755
KIRC	269 (< = 60)	270 (>60)	0.17	353	186	0.197
KIRP	133 (< = 60)	153 (>60)	0.132	212	77	0.351
LAML	88 (< = 60)	63 (>60)	0.027	83	68	0.944
LGG	264 (< = 40)	264 (>40)	<0.001	289	239	< 0.001
LIHC	87 (< = 177)	81 (>196)	0.044	253	121	0.01
LUAD	255 (< = 65)	261 (>65)	0.559	249	286	0.683
LUADLUSC	446 (< = 65)	563 (>65)	0.111	620	417	0.846
LUSC	191 (< = 65)	302 (>65)	0.089	371	131	0.98
MESO	47 (< = 65)	39 (>65)	0.226	71	15	0.762
OS	78 (< = 18)	23 (>18)	0.494	60	41	0.133
OSCC	155 (< = 60)	173 (>60)	0.863	227	102	0.014
OV	208 (< = 60)	171 (>60)	0.475	-	-	-
PAAD	93 (< = 65)	85 (>65)	0.226	98	80	0.181
PCPG	108 (< = 50)	75 (>50)	0.879	81	102	0.199
PRAD	224 (< = 60)	275 (>60)	0.856	-	-	-
READ	82 (< = 65)	84 (>65)	0.906	91	75	0.361
SARC	130 (< = 60)	133 (>60)	0.267	119	144	0.011
SKCM	252 (< = 60)	211 (>60)	0.186	292	179	0.314
STAD	164 (< = 65)	207 (>65)	0.548	241	134	0.945
TGCT	67 (< = 30)	72 (>30)	0.838			
THCA	241 (< = 45)	269 (>45)	0.067	139	371	0.315
THYM	6 (< = 60)	57 (>60)	<0.001	62	57	0.645
UCEC	206 (< = 60)	303 (>60)	0.057	-	-	-
UCS	22 (< = 65)	34 (>65)	0.682	-	-	-
UVM	40 (< = 60)	40 (>60)	0.609	45	35	0.621

### GSEA of *BCL7B*

**FIGURE 6:**
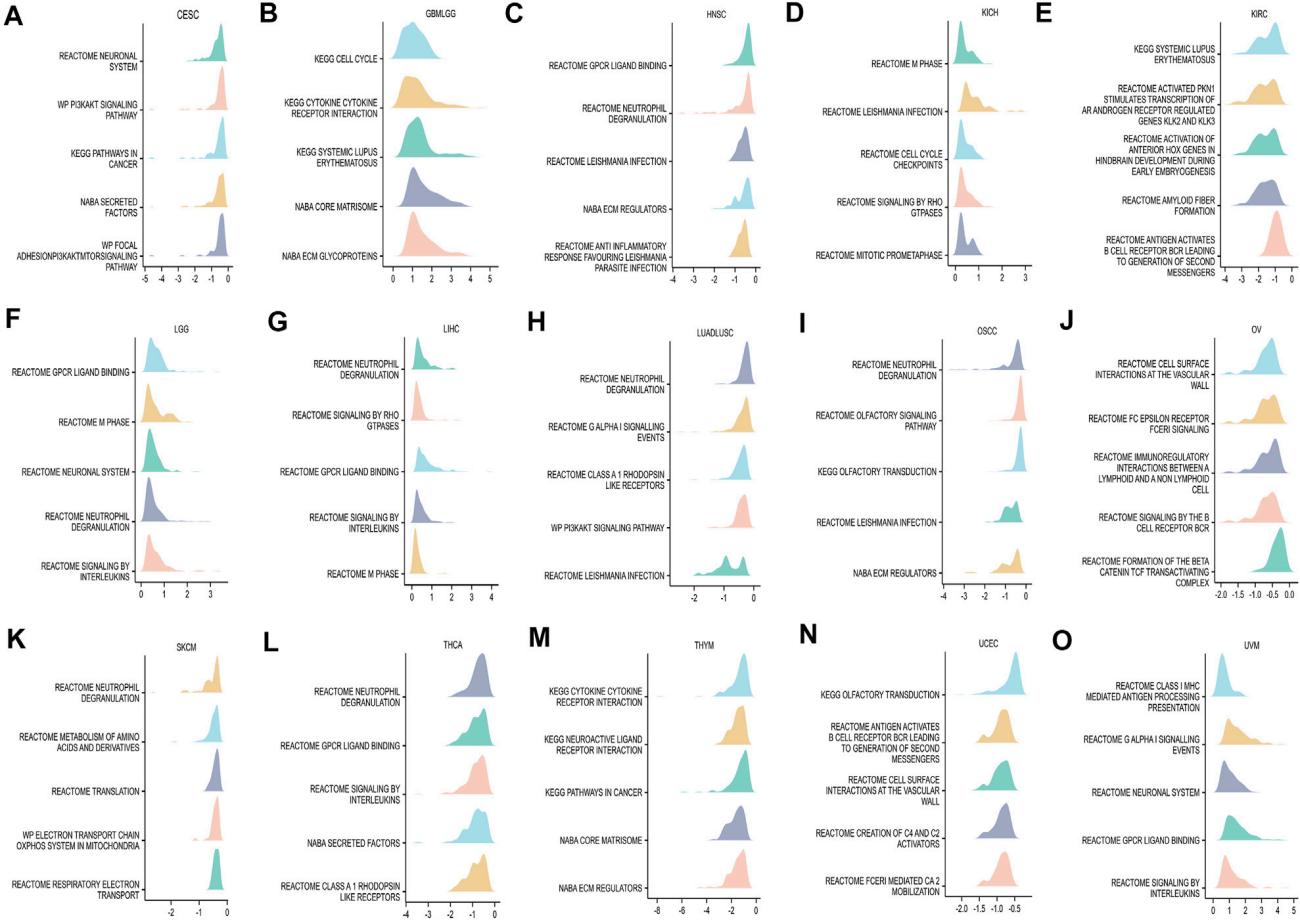
GSEA of *BCL7B* in pan-cancer. **(A–O)** Top five GSEA terms in indicated tumor types.

GSEA was conducted to explore the top five GSEA terms through which *BCL7B* may involve in 33 tumor types from TCGA. The results suggested that *BCL7B* was notably associated with cancer-related and immune-related pathways, especially for immunoregulatory interaction between a lymphoid and a non-lymphoid, antigen activates B-cell receptor BCR leading to generation of secondary messengers, neutrophil degranulation, pathways in cancer, cell cycle checkpoints, and signaling by interleukins, such as in CESC, GBMLGG, HNSC, KICH, KIRC, LGG, LIHC, LUADLUSC, OSCC, OV, SKCM, THCA, THYM, UCEC, and UVM (Figures 6A–O). But the aforementioned pathways were not observed in other tumor types (Supplementary Figure 2).

### Correlation Analysis of Immune-Activating and -Suppressing Genes and DNA Repair Genes

**FIGURE 7:**
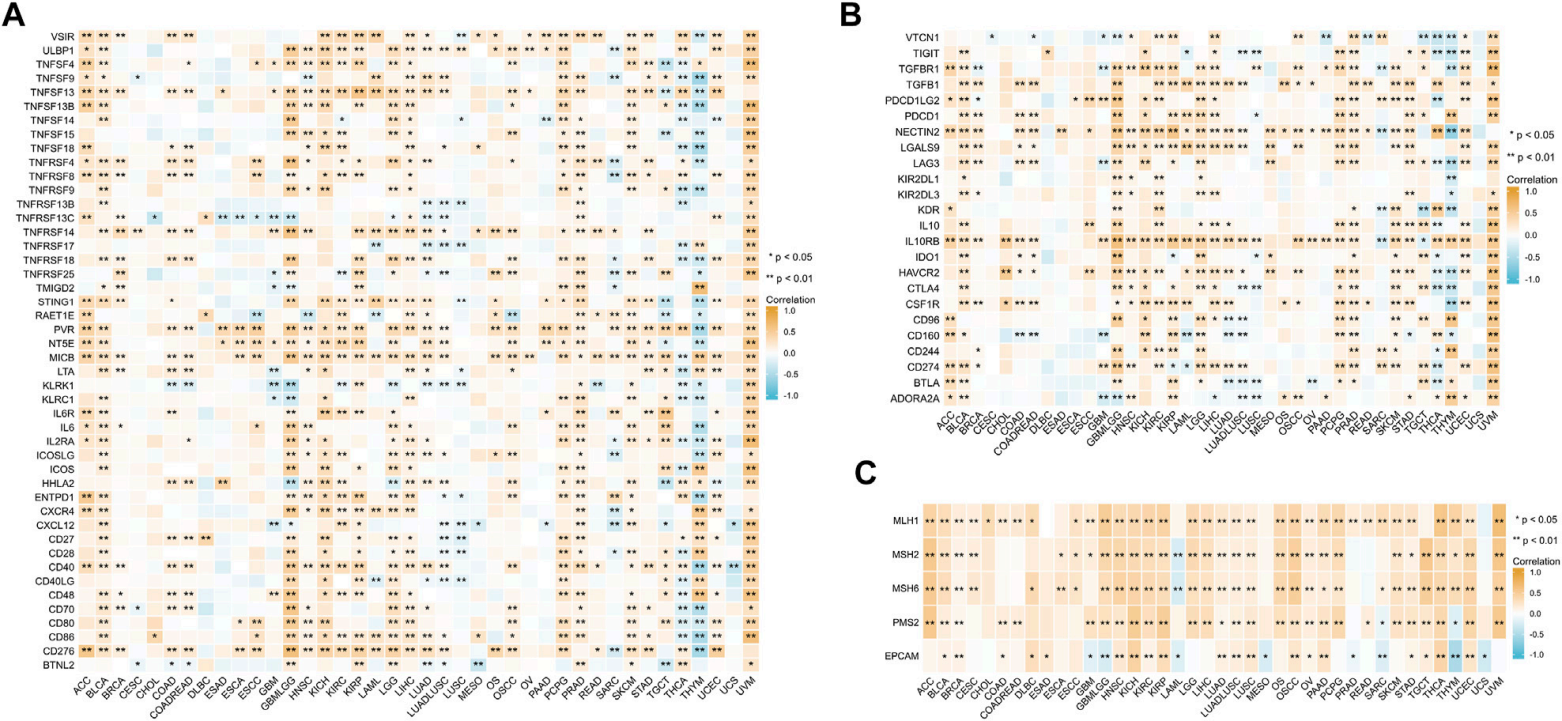
Correlation between *BCL7B* gene expression and immune-activating genes **(A)**, immune-suppressing genes **(B)**, and DNA repair genes **(C)**. ∗*p* < 0.05 and ∗∗*p* < 0.01.

To further explore the role of *BCL7B* gene in the immune process and DNA repair, Spearman’s rank correlation coefficient was used to analyze the relationships between *BCL7B* gene expression and immune-activating and -suppressing genes and DNA repair genes. The results suggested that *BCL7B* had varying degrees of correlation with 46 immune-activating genes (Figure 7A), 24 immune-suppressing genes (Figure 7B), and 5 DNA repair genes (Figure 7C) in 40 types of tumors.

### Associations of *BCL7B* and Immune Subtypes of Cancers

To further investigate whether *BCL7B* potentially affects immune subtypes (Immune Landscape) of human cancers, this study also explored the associations between *BCL7B* expression and immune subtypes in human pan-cancer. The results showed that *BCL7B* expression was significantly different across immune subtypes in 10 cancer types (Supplementary Figure 3).

### Immunohistochemical Staining of *BCL7B* in the Human Protein Atlas Database

**FIGURE 8:**
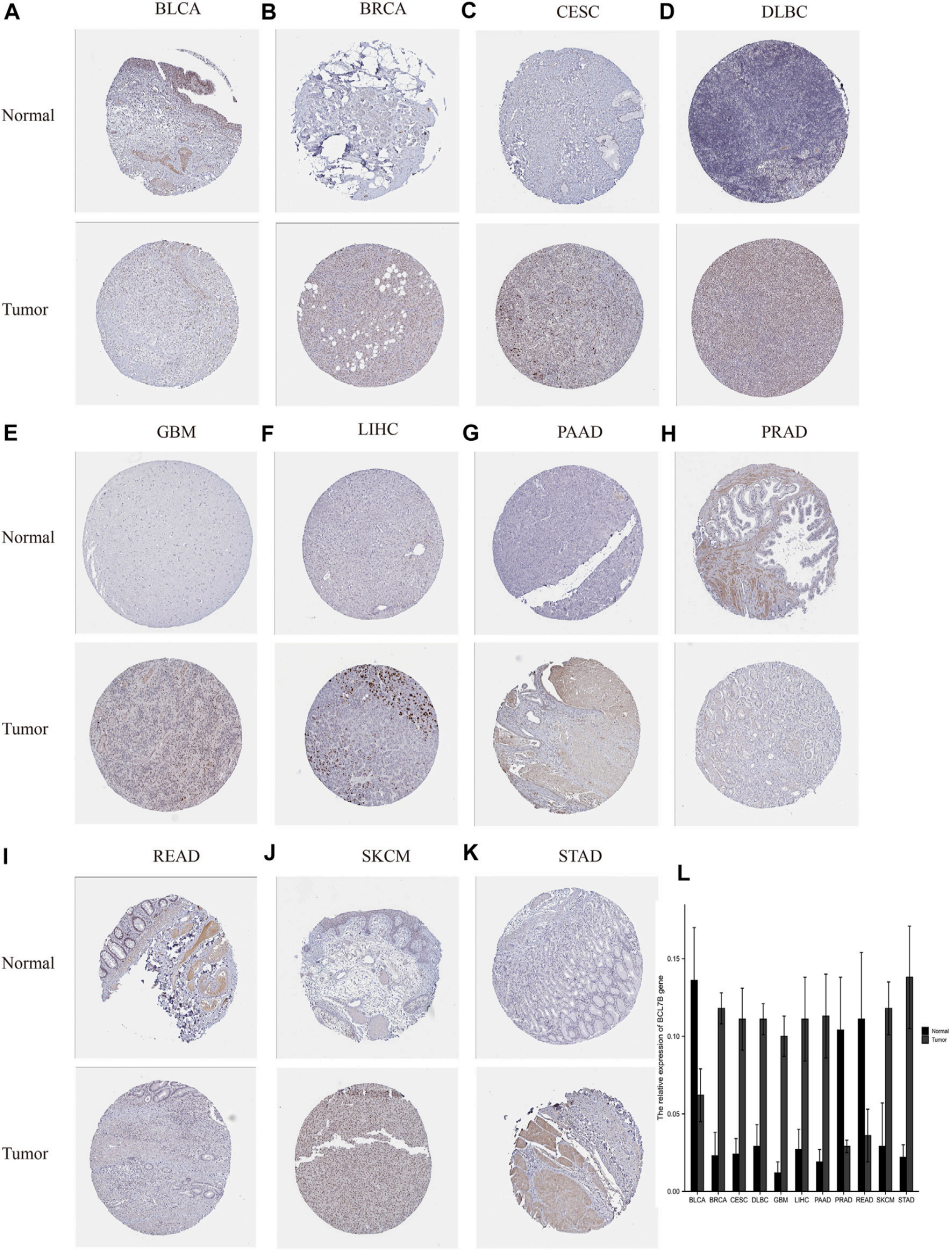
Verification of *BCL7B* gene expression in tumors and corresponding normal tissues based on the HPA database. At the protein level, *BCL7B* gene expression was high in tumor tissues in BRCA, CESC, DLBC, GBM, LIHC, PAAD, SKCM, and STAD **(B–G, J,K)** and low in BLCA, PRAD, and READ **(A,H,I)**. **(L)** Quantitative expression of *BCL7B* gene.

To further verify the protein expression of *BCL7B* gene in 33 tumor tissues and corresponding normal tissues, and to make our results more convincing, we explored the immunohistochemical staining of *BCL7B* in the HPA database. Our results confirmed that, at the protein level, *BCL7B* was significantly highly expressed in BRCA, CESC, DLBC, GBM, LIHC, PAAD, SKCM, and STAD and lowly expressed in BLCA, PRAD, and READ compared with the corresponding normal tissues (Figure 8).

## Discussion

Significant efforts have been made in analyzing oncogenic pathways across human cancers. However, the pathological process of cancers results from complex interplay. Accumulative evidence showed that the differential gene expression could contribute to the initiation and progression of malignant tumors. DNA methylation is also an important regulator of gene transcription and expression as epigenetic alterations, and the aberrant methylation of DNA is the key contributing to cancer development. Innate immunity involves various types of myeloid lineage cells, including DC, monocytes, macrophages, polymorphonuclear cells, mast cells, and innate lymphoid cells (ILCs) such as NK cells (Vivier et al., 2018; Demaria et al., 2019). Cancer cells highly express the immune inhibitory signaling proteins, which directly affect the function of immune cells. Novel cancer immunotherapy is the most promising cancer treatment strategy, mainly including chimeric antigen receptor T cell and immune checkpoint inhibitors (Yi et al., 2018). The key role of T cells in tumor immunity has been demonstrated by the positive correlation between prognosis and the T-cell infiltration at tumor bed (Pagès et al., 2018). Immune checkpoints have distinct ligands expressed on T cells and suppress T-cell function through multiple mechanisms. CTLA-4 interacts with CD80/CD86, thereby limiting T-cell activation and leading to anergy. PD-1 interacted with PD-L1 expressed on antigen-presenting cells (APCs), and tumors send a negative signal to T cells, which leads to T-cell exhaustion (Dyck and Mills, 2017). Given their role in suppressing effector T-cell responses, immune checkpoints are targeted for the treatment of cancers. However, the response rate of immune checkpoint inhibitors in overall patients is unsatisfactory, which limits the application in clinical practice. Hence, it is necessary to study the new suppression checkpoints and their target molecules to expand the efficacy of immune therapy. Recent studies have shown that epigenetic regulation affects all cancer hallmarks in all aspects of the interaction between tumor cells and the immune system (Cao and Yan, 2020).

A number of studies have found that BCL7 family members are involved in tumorigenesis and progress. It is shown that *BCL7B*-mediated dephosphorylation of cAMP response element binding protein (CREB) could regulate the formation of membrane protrusions, resulting in PAAD cell motility and invasion (Taniuchi et al., 2018). Previous studies showed that the *BCL7B* gene regulates the apoptotic and Wnt signaling pathways, which involve in tumor suppression (Uehara et al., 2015). Little is known about the role of *BCL7B* in malignancies. Understanding the extent and detailed landscape of *BCL7B* function is important for researchers that focused on the pathogenesis and development of cancers. Here, we performed a systematic analysis and provided a complete picture of *BCL7B* function in human cancers. To the best of our knowledge, this is the first comprehensive and bioinformatic analysis of *BCL7B* complexes revealing extensive roles and related mechanisms across human malignancy.

Previous studies have revealed the genetic backgrounds of certain types of cancers, for example, the MSH2 gene in familial nonpolyposis colon cancer, the BRCA gene in familial breast cancer, the APC gene in colorectal cancer, and the RB gene in retinoblastoma (Golabchi et al., 2018; Aghabozorgi et al., 2019; Tamura et al., 2019; Venkitaraman, 2019). Differential gene expression is involved in cancer development and patient survival, but only few gene markers have been discovered so far. It is necessary to detect more biomarkers that play essential roles in cancer progression. We first assessed the expression of *BCL7B* gene in 40 normal and tumor tissues from TCGA dataset and found that its expression was higher in eight tumors, including BRCA, CHOL, ESCA, GBM, HNSC, KIRC, KIRP, and LIHC. The *BCL7B* gene expression was lower in seven tumors including BLCA, KICH, LUAD, LUSC, PRAD, READ, and THCA. To get more data of normal control tissues, we also explored the GTEx database and combined it with TCGA database. Differential expression of *BCL7B* between tumor and normal tissues existed in more types of cancers. The analysis showed that the expression of *BCL7B* was lower in BCLA, COAD, ESCA, LUAD, LUSC, PRAD, READ, THCA, UCEC, and UCS. The expression of *BCL7B* gene was higher in ACC, CESC, CHOL, DLBC, GBM, HNSC, KIRC, KIRP, LGG, LIHC, OV, PAAD, SKCM, STAD, and THYM. Furthermore, the HPA database verified the protein expression information of *BCL7B* by immunohistochemistry in 11 types of cancers including BRCA, CESC, DLBC, GBM, LIHC, PAAD, SKCM, STAD, BLCA, PRAD, and READ.

We also explored the association between the *BCL7B* expression level and patient prognosis in several cancers. In KIRC, SARC, KIRP, SKCM, and THCA, the decreased level of BLC7B predicted poor OS, while high *BCL7B* expression was positively correlated with OS in GBM, GBMLGG, KICH, LGG, OSCC, UVM, and READ patients. For DSS, the results revealed that *BCL7B* acts as a protective factor for patients with KIRP and SARC, and a risk factor for patients with GBM, GBMLGG, LGG, and READ. However, the use of OS and DSS as endpoints does not necessarily reflect tumor progress or response to treatment. In addition, using DSS or OS requires longer follow-up time. Hence, the use of PFI could more effectively reflect the impact of factors on patients. So, we further performed Spearman’s rank correlation coefficient analysis to assess the association between *BCL7B* gene expression and PFI of tumor patients. It was found that high expression of *BCL7B* gene was correlated with inferior PFI in GBM, GBMLGG, KICH, LGG, OSCC, UVM, and READ. In contrast, the downregulated expression was associated with poor PFI in KIRC, SARC, KIRP, SKCM, and THCA. Our study illustrated the protective and carcinogenic role of *BCL7B* in cancer patients. Above all, the findings indicated that *BCL7B* expression could be used as a predictor of tumor prognosis. This highlighted that it is more feasible to predict the prognosis of cancer patients by considering the combination of multiple data.

At present, identified diagnosis is relied on invasive pathology. Moreover, glycoprotein cancer biomarkers such as prostate-specific antigen (PSA), carcinoembryonic antigen (CEA), and SLe tetrasaccharide (CA199) serve widely as prognostic factors and response assessment to therapy (Hristova and Chan, 2019). Discovery of cancer biomarkers most frequently originates in academic research settings, leading to the identification of thousands of proteins and genetic markers. In our study, the ROC curves revealed that *BCL7B* gene was of great diagnostic value in DLBC, ESAD, HNSC, OSCC, OV, PAAD, SKCM, GBM, and THYM. *BCL7B* may contribute to the noninvasive diagnosis of the aforementioned cancers.

Our results revealed the relationship between immune infiltration and *BCL7B* gene expression in immune microenvironment. We analyzed the expression of *BCL7B* and six types of infiltrating immune cells (B cells, dendritic cells, neutrophils, T cells, macrophages, and NK cells) in 39 tumors. We found that only BLCA, LGG, PRAD, and THCA had correlation with all the aforementioned six immune cells. We found that the *BCL7B* gene expression was positively correlated with the six types of infiltrating immune cells in BLCA, which conformed to the strong correlation between tumor immune infiltration and BLCA (Ding et al., 2021). A more in-depth research based on tumor samples revealed that the *BCL7B* gene expression has different degrees of correlation with infiltrating immune cell subsets in a multiple tumor environment. Malignant tumor tissues not only include tumor cells but also tumor-associated normal epithelial, stromal, immune, and vascular cells. Stromal and immune cells are the main components of normal cells in tumor tissues (ESTIMATEScore is the sum of stromal and immune score). Furthermore, the expression of the *BCL7B* gene was correlated with ESTIMATEScore in BLCA, BRCA, ESCC, GBMLGG, LAML, LGG, LUADLUSC, LUSC, PAAD, PRAD, UCEC, and UVM. In this stratified medicine era, identifying new immune-related biomarkers is increasingly vital. Our findings contribute to the identification of new immune-related therapeutic targets (Chen et al., 2019).

Immune escape is one of the initial steps of metastasis. It is crucial for diverse steps of metastasis including the onset, dissemination, survival of tumor, and eventually reaching new organs. Human T cells, when activated, express immune checkpoint proteins, including programmed cell death protein 1 (PD-1) and cytotoxic T lymphocyte-associated antigen-4 (CTLA-4), which negatively regulate T-cell function (Arasanz et al., 2017; Khailaie et al., 2018). In particular, cancer cells bind to and activate negative molecules on the surface of T cells to help secrete soluble immunosuppressive media into the microenvironment. T-cell receptor (TCR) engagement with an antigen presented *via* the major histocompatibility complex (MHC) requires activation of costimulatory second signal delivered by CD28. CTLA-4 is a competitive CD28 homolog that binds CD28 ligands CD80/86, preventing T-cell activation (Willsmore et al., 2021). The engagement of PD-1 by its ligands results in the recruitment of Src homology 2 (SH2) domain containing phosphatases 1/2 (SHP1/2) and then inhibits T-cell proliferation and cytokine secretion mediated by TCR (Dermani et al., 2019; Qin et al., 2019). Antibody-based therapies targeting CTLA-4 and PD-1 have achieved lasting responses in some patients against a range of cancer types. The therapy of immune checkpoint blockade in patients with a wide variety of malignancies has made great progress in recent years (Patel and Minn, 2018). Our result reflected that *BCL7B* had different degrees of correlation with 47 immune checkpoints in tumor microenvironment.

TMB is the number of somatic mutation apart from germline mutation in tumor genomes. MSI reflects the degree of defective DNA mismatch repair (dMMR) in tumor cells. MSI and dMMR represent the subgroup of malignancies with novel therapeutic opportunities (Le et al., 2015). The higher TMB and MSI means more new antigens produced by tumor cells, which can be recognized as nonself by immune cells and triggers antitumor immune response (Rizvi et al., 2015; Addeo et al., 2021). In general, TMB and MSI are significant biomarkers for predicting immunotherapy efficacy. We revealed the obvious correlation between *BCL7B* gene expression and TMB, and MSI in multiple tumors such as PRAD, UCEC, and SARC. These findings contribute to understanding the special role of *BCL7B* in prediction of immunotherapy and prognosis.

Bioinformatic enrichment analysis showed that *BCL7B* was notably associated with the interaction pathways of immunoregulatory, cytokine–cytokine receptor interaction, and neutrophil degranulation in CESC, GBMLGG, HNSC, KICH, KIRC, LGG, LIHC, LUADLUSC, OSCC, OV, SKCM, THCA, THYM, UCEC, and UVM. In particular, *BCL7B* was significantly related to the pathways in cancer in CESC and THYM. Cytokines play essential roles in the development, differentiation, and function of myeloid and lymphoid cells. Dysregulated cytokine expression can activate the janus kinase (JAK)/signal transducer and activator of transcription (STAT) pathway in all human cancers (Chikuma et al., 2017; Propper and Balkwill, 2022). Neutrophils are believed to be a crucial component of the chronic inflammation process, which are well-recognized as a major hallmark of cancer. In the cancer setting, the excessive release of neutrophil granules may regulate tissue microenvironment, which ultimately leads to tumor initiation and metastasis (Rawat et al., 2021). The results highlight the potential mechanism of *BCL7B* in the progress of different cancer types.

Abnormal DNA methylation patterns are extensively involved in tumor development. Evidence has showed that aberrant DNA methylation is a typical hallmark of cancers (Ibrahim et al., 2022). Pan-cancer methylation patterns reveal common mechanisms and new similarities of *BCL7B* in different cancers (Shi et al., 2020). We surveyed the *BCL7B* on DNA methylation patterns in normal or tumor states to illustrate their potential roles in pan-cancer. Our result revealed that the *BCL7B* gene expression is positively correlated with DNA methylation in BLCA, LIHC, PRAD, SARC, and THYM and negatively in HNSC, KIRP, LUAD, LUADLUSC, PCPG, TGCT, and THCA. In addition, *BCL7B* gene expression was closely correlated with clinical features, including age, gender, and TNM stages. This may help to assess clinical severity in patients with tumors.

However, our work is a retrospective study based on public databases. Follow-up animal experiment verification and further multicenter, large-sample, prospective studies are required to testify the relationship between *BCL7B* and patient prognosis, to explore the role of *BCL7B* in cancer progress and to seek more effective treatment strategies. For example, immunohistochemistry (IHC) and flow cytometry can be used to explore the immune infiltration in tumor tissues by detecting the markers of immune cells (Shrestha Palikhe et al., 2021; Wang et al., 2022). Cell counting kit-8 (CCK-8) and transwell tests can be used to detect the proliferation and invasion of tumor cells in *BCL7B* high and low expression groups (Liu et al., 2021; Zhang et al., 2021). The clinical application of *BCL7B* for diagnosis still requires more support of basic and clinical researches.

In summary, our systematic analysis provided a novel insight into the *BCL7B* expression, prognostic significance and the relationship between *BCL7B* expression and immune cell infiltration, immune checkpoints, DNA methylation, DNA repair genes, TMB, and MSI in pan-cancer. These analyses contribute to elucidate the role of *BCL7B* in tumor diagnosis, prognosis, and tumorigenic mechanism.

## Data Availability

The original contributions presented in the study are included in the article/Supplementary Material. Further inquiries can be directed to the corresponding author.
