# Supplementary figures and images for: A Pan-Cancer Analysis of the Oncogenic Role of BCL7B: A Potential Biomarker for Prognosis and Immunotherapy
Source: Front Genet. 2022 Jul 15;13:906174. doi: 10.3389/fgene.2022.906174 (PMC9334570; doi:10.3389/fgene.2022.906174)

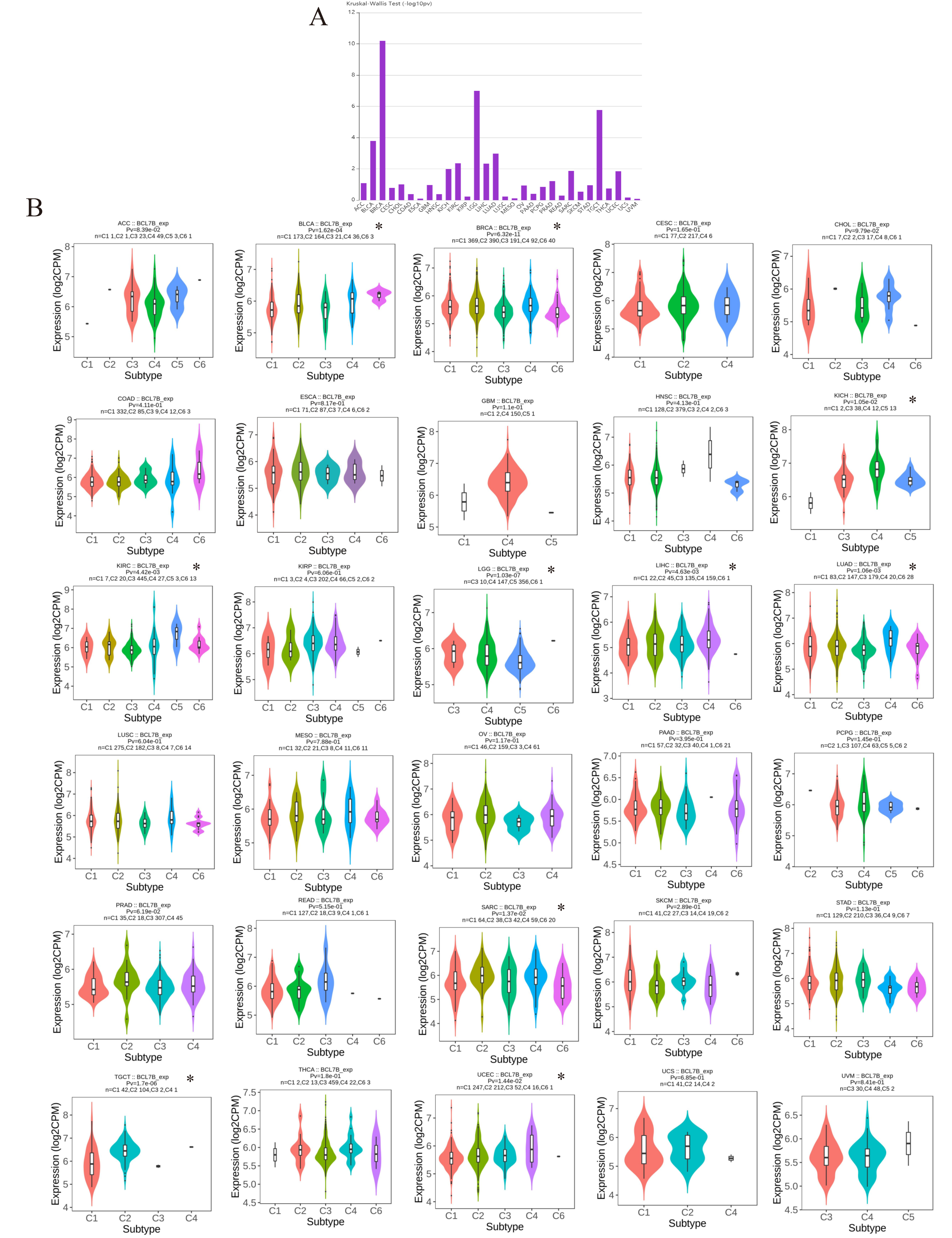

Supplement: Supplementary file 1 [file Image3.TIF]

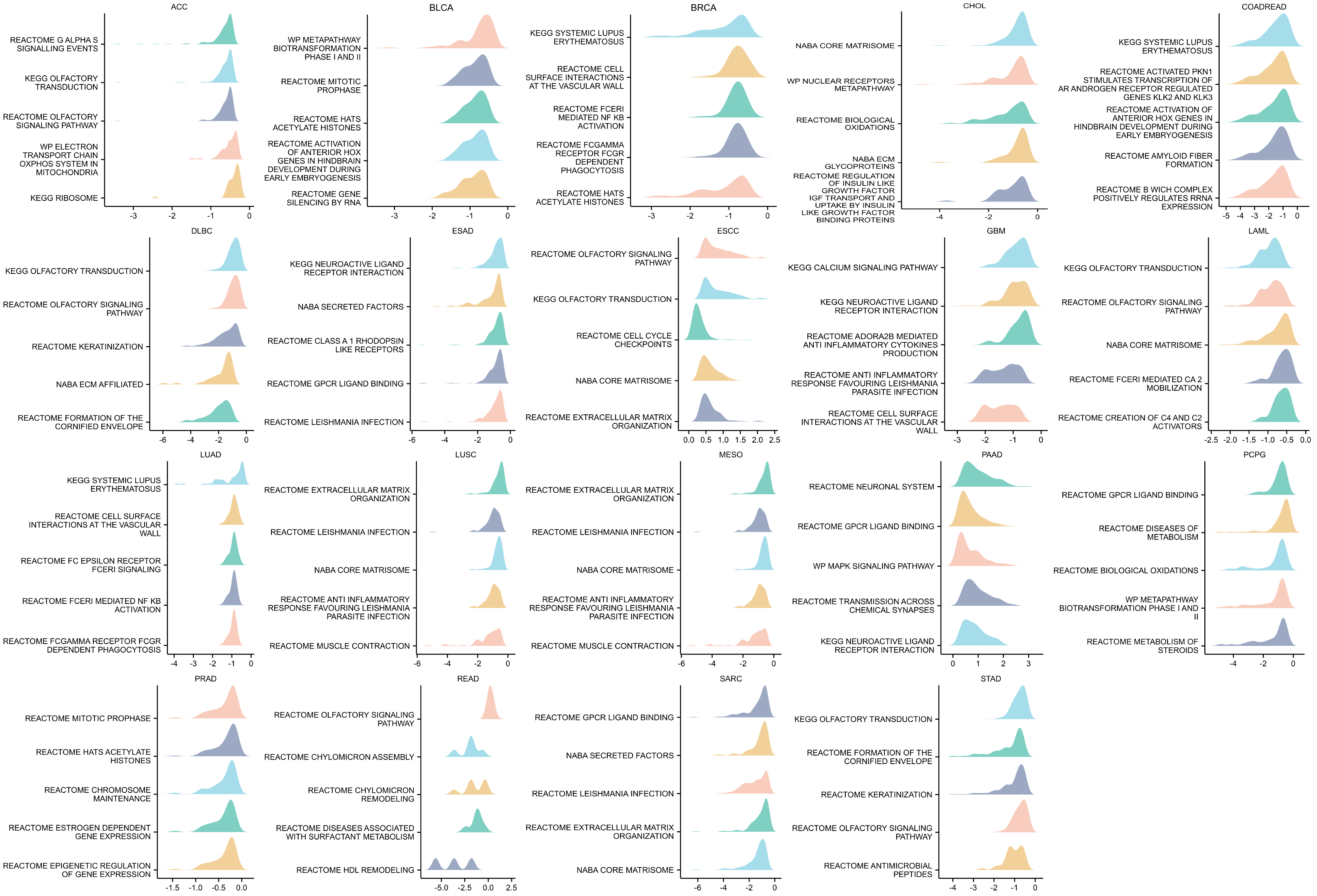

Supplement: Supplementary file 2 [file Image2.TIF]

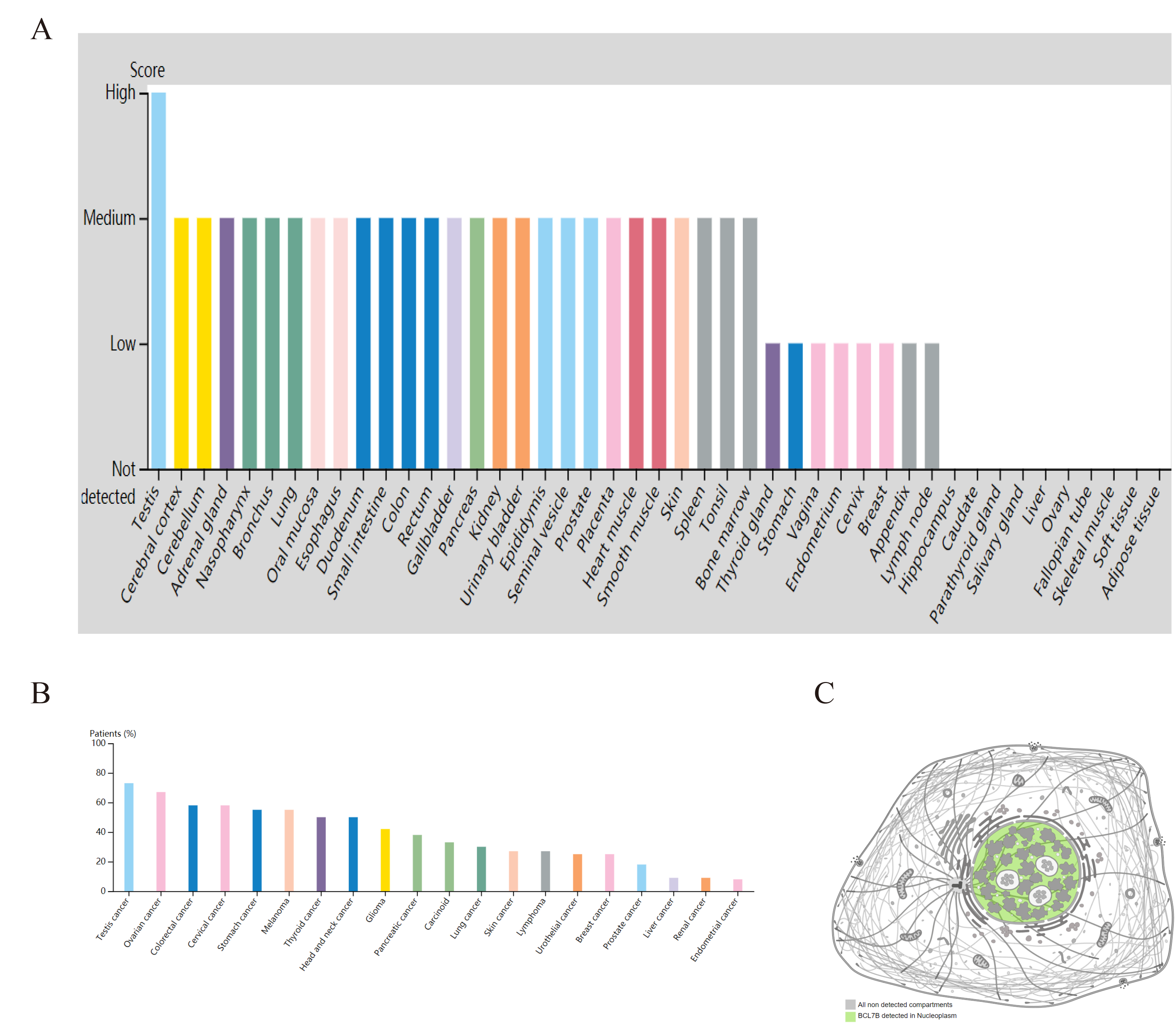

Supplement: Supplementary file 3 [file Image1.TIF]
